# Phase II trial of short-term neoadjuvant docetaxel and complete androgen blockade in high-risk prostate cancer

**DOI:** 10.1038/sj.bjc.6605320

**Published:** 2009-09-15

**Authors:** B Mellado, A Font, A Alcaraz, L A Aparicio, F J G Veiga, J Areal, E Gallardo, N Hannaoui, J R M Lorenzo, A Sousa, P L Fernandez, P Gascon

**Affiliations:** 1 Medical Oncology Department, Hospital Clinic, Barcelona, Spain; 2 Medical Oncology Department, Hospital Germans Trias I Pujol, Catalan Institute of Oncology, Badalona, Spain; 3 Urology Department, Hospital Clinic, Barcelona, Spain; 4 Medical Oncology Department, Hospital Juan Canalejo, La Coruña, Spain; 5 Urology Department, Hospital Juan Canalejo, La Coruña, Spain; 6 Urology Department, Hospital Germans Trias I Pujol, Badalona, Spain; 7 Medical Oncology Department, Hospital Parc Tauli, Sabadell, Spain; 8 Urology Department, Hospital Parc Tauli, Sabadell, Spain; 9 Medical Oncology Department, Complejo Xeral Calde, Lugo, Spain; 10 Urology Department, Hospital Monforte de Lemos, Monforte de Lemos, Lugo, Spain; 11 Pathology Department, Hospital Clinic, Barcelona, Spain

**Keywords:** prostate cancer, high risk, neoadjuvant therapy, docetaxel

## Abstract

**Background:**

The low probability of curing high-risk prostate cancer (PC) with local therapy suggests the need to study modality of therapeutic approaches. To this end, a prospective phase II trial of neoadjuvant docetaxel (D) and complete androgen blockade (CAB) was carried out in high-risk PC patients. The primary end point was to detect at least 10% of pCRs after chemohormonal treatment.

**Methods:**

Patients with T1c–T2 clinical stage with prostate-specific antigen (PSA) >20 ng ml^−1^ and/or Gleason score ⩾7 (4+3) and T3 were included. Treatment consisted of three cycles of D 36 mg m^−2^ on days 1, 8 and 15 every 28 days concomitant with CAB, followed by radical prostatectomy (RP).

**Results:**

A total of 57 patients were included. Clinical stage was T1c, 11 patients (19.3%); T2, 30 (52.6%) and T3, 16 (28%) patients. Gleason score was ⩾7 (4+3) in 44 (77%) patients and PSA >20 ng ml^−1^ in 15 (26%) patients. Treatment was well tolerated with 51 (89.9%) patients completing neoadjuvant therapy together with RP. The rate of pCR was 6% (three patients). Three (6%) additional patients had microscopic residual tumour (near pCR) in prostate specimen. With a median follow-up of 35 months, 18 (31.6%) patients presented PSA relapse.

**Conclusion:**

Short-term neoadjuvant D and CAB induced a 6% pCR rate, which is close to what would be expected with ADT alone. The combination was generally well tolerated.

Patients with high-risk localised or locally advanced prostate cancer (PC) are defined by prostate-specific antigen (PSA) >20 ng ml^−1^, Gleason score >7 and clinical stage T3/T4. In this group of patients, reported rates of disease-free survival (DFS) after local therapy range from 30 to 50% (D'Amico, 2007; Joniau and Van Poppel, 2008). In this context, the role of systemic adjuvant or neoadjuvant therapy has been investigated with the aim of improving the results obtained with local therapy alone.

In this regard, the use of early and prolonged androgen ablation in association with radiation therapy delays disease progression and improves overall survival. Randomised trials have also shown that long-term hormonal therapy is superior to short-term hormonal therapy in association with radiation therapy. However, long-term androgen suppression is associated with an increased risk of toxicity (Hanks *et al*, 2003; D'Amico *et al*, 2008; Roach *et al*, 2008; Sanda *et al*, 2008). In contrast, the use of neoadjuvant hormonal therapy alone before prostatectomy has shown reduction of positive surgical margins but results in anecdotal pCRs. Moreover, no benefit to patient outcome was observed in randomised trials of neoadjuvant hormonal therapy and no differences between short- and long-term treatments were reported (Kumar *et al*, 2006).

More recently, on the basis of the evidence that docetaxel (D) has clinical activity and increases survival in patients with metastatic hormone refractory PC (Petrylak *et al*, 2004; Tannock *et al*, 2004; Berthold *et al*, 2008), this drug has been tested as neoadjuvant therapy in high-risk localised PC. In neoadjuvant studies including a limited number of patients and using different chemotherapy schedules, D alone had clinical activity but no pCRs were reported (Dreicer *et al*, 2004; Febbo *et al*, 2005; Magi-Galluzzi *et al*, 2007).

In this study, we sought to study the potential role of the combination of chemotherapy and hormonal treatment in a neoadjuvant setting. In particular, we explored the combination of D and hormonal therapy with the objective of targeting both the androgen-dependent and -independent cell subpopulations that may coexist at the primary tumour and/or micrometastatic disease. We report here the activity of weekly D in combination with short-term (3 months) CAB before radical prostatectomy (RP) in high-risk localised PC patients. We chose this schedule on the basis of the low toxicity profile of weekly D and short-term CAB, and because the duration of neoadjuvant therapy would not significantly delay local therapy. The main objective of the study was to determine the rate of pCR of the combined therapy.

## Patients and methods

### Study design

The study was a multicentre non-randomised phase II trial. The main objective was to determine the percentage of pCR achieved with D together with complete androgen blockade (CAB) in patients with high-risk PC. Secondary objectives were to determine the toxicity and clinical activity of the study combination. The study was approved by the Spanish Ministry of Health; and by the internal review board and ethics committee of each participating institution.

### Patients

Inclusion criteria were histologically confirmed adenocarcinoma of the prostate with any of the following risk criteria: (1) clinical stage T3 or (2) clinical stage T1c or T2 with serum PSA >20 ng ml^−1^ and/or Gleason score sum of 8, 9 or 10; or a Gleason sum of 7 with a predominant form of 4 (i.e. Gleason score 4+3=7). Patients had to be suitable candidates for RP. An Eastern Cooperative Oncology Group performance status of 0–1 was required. Normal blood cell count and biochemistry were required. Patients had to sign an informed consent to be included in the study.

### Treatment and monitoring

Patients were treated with D 36 mg m^−2^ on days 1, 8 and 15 every 28 days, for three cycles, together with CAB consisting of flutamide, 250 mg p.o. three times per day, starting day 1, for 12 weeks and trimestral goserelin, a single s.c. dose of 10.8 mg on day 15. Pre-medication with oral dexamethasone consisted of 8 mg dose 1 h before every D administration. Anti-emetics were allowed at the discretion of the treating physician. Baseline study consisted of clinical anamnesis, complete blood count and biochemistry, serum testosterone, physical exam, digital rectal examination, endorectal ultrasound, thorax X-ray, bone gammagraphy, and abdominal and pelvic CT scan or MRI. Patients were assessed for toxicity on days 1, 8 and 15 of every cycle, before chemotherapy administration. Patients were allowed one dose-level reduction of D for toxicity. Every 4 weeks, digital rectal examination, complete blood cell count and chemistries, serum testosterone and PSA level measurement were repeated for all the patients. Pelvic CT scan or MRI was repeated before RP. If severe toxicity or disease progression were documented during the treatment period, patients were withdrawn from the study and were offered local therapy with RP or radiotherapy. In patients who completed neoadjuvant therapy, RP was performed 2–4 weeks after the end of systemic therapy. The administration of immediate postoperative radiation therapy was allowed in patients with ⩾pT3 and/or positive surgical margins. Adjuvant hormonal therapy was allowed in case of histologically confirmed lymph node involvement. After surgery, patients were followed with clinical anamnesis and PSA every 3 months during the first 2 years, every 6 months during the next 3–5 years and annually thereafter. Prostate-specific antigen response was evaluated before RP, following the PSA response criteria (Bubley *et al*, 1999). Prostate-specific antigen relapse was considered as a PSA >0.4 nm ml^−1^ after RP, confirmed in at least two determinations (Heidenreich *et al*, 2008).

### Pathologic evaluation

All pathologists followed the same procedures to assess pathologic response to chemotherapy. Processing and analysis of tumour samples were standardised for all participating centres. Multiple sections of the whole surgical specimen were processed. A pCR was defined as complete eradication of the tumour, evidenced by the absence of residual neoplastic cells in the surgical specimen determined by haematoxylin and eosin staining. We considered that the postoperative Gleason score may be altered by the effects of neoadjuvant therapy and that its evaluation may differ by pathologist. For that reason, we did not report these data. Frozen and paraffin-embedded prostatectomy samples were prospectively harvested for future correlative scientific studies.

### Statistical analysis

Considering that the reported percentages of pCR obtained with 3-month hormonal therapy alone range from 0 to 4%, we chose as the primary end point a 10% or more pCR rate after chemohormonal treatment. The sample size was calculated by the Gehan method (*α* error of 0.05 and *β* error of 10%) (Hanfelt *et al*, 1999). A three-stage design was specified. If at least one pCR was observed in the first 22 patients included, the study would enrol an additional 20 patients. If at least two pCRs were observed, the sample size would again be increased up to 57 patients. Median time to PSA progression, clinical progression and survival was considered since the time of inclusion in the study until PSA progression, clinical progression or death occurred, and was calculated by the Kaplan–Meier method (Kaplan and Meier, 1975).

## Results

### Patient characteristics

From August 2004 to July 2006, 57 patients were included in the study. Patient characteristics are shown in Table 1. All patients were node negative and free from metastatic disease by imaging studies. Median age was 66 (range, 66–68.3). Clinical stage was T1c in 11 patients (19.3%), T2 in 30 (52.6%) and T3 in 16 (28%) patients. The Gleason score was ⩾7 (4+3) in 44 (77%) patients, and PSA was >20 in 15 (26%) patients. Median PSA was 9.7 (range, 0.6–90.8). Thirty-three patients (58%) presented one risk factor, 21 (36%) two and 3 (5%) had three risk factors.

### Neoadjuvant therapy

A total of 161 cycles of D were administered, with a median of three cycles per patient. Four patients received one chemotherapy cycle and two patients received two cycles. The remaining patients completed the planned therapy. Dose reduction of D was performed in 10 patients (17.5%). In 15 patients (26.3%) flutamide was discontinued. All patients received the planned single dose of goserelin. Median relative dose intensity was 96% for D and 90% for flutamide. Toxicities are shown in Table 2. Grades 3 and 4 toxicities were diarrhoea and liver abnormalities, mainly attributed to flutamide. Flutamide was chosen because it was the most widely used anti-androgen by the investigators in clinical practice at the time of trial design. In the light of the observed hepatotoxicity with the combination of D and flutamide in this study, we suggest that further combination studies should consider replacing flutamide with other anti-androgens that have fewer liver side effects.

Prostate-specific antigen response was evaluated in 51 of 57 patients after chemo-hormonal therapy and before RP. One patient experienced PSA stabilisation, 24 (47%) had partial PSA response and in 26 (51%) PSA became undetectable. None of the patients had PSA progression during neoadjuvant therapy. The PSA response rate was 93% (range, 86–99%, with a 95% CI).

### Surgery

A total of –51 (89.9%) patients completed the three cycles of D and hormone therapy and underwent RP. Bilateral pelvic lymphadenectomy was performed in 49 of the 51 patients who underwent RP. Median number of extracted lymph nodes was 6 (range, 1–15). Five patients did not complete the planned neoadjuvant treatment because of toxicity (one underwent RP and the rest were treated with radiation therapy plus hormonal therapy). One patient refused surgery after completing the neoadjuvant therapy. Of the 51 patients who underwent RP, 1 experienced a urinary fistula. No other major surgical complications were observed.

### Pathological response

Of the 51 patients evaluated for pathological response, in 33 (64.7%) the surgical margins were negative and in 27 (53%) no extraprostatic involvement was observed. Lymph node involvement was detected in two (3.9%) patients. In three (6%) patients, no evidence of tumour cells was detected in prostatectomy specimens. They were counted as having a pCR. In three additional patients (6%), there was only residual microscopic tumour (Table 3). Pathological stage was pT0 in 3 patients (5.9%), pT2 in 29 (57%) and pT3 in 19 (37%) patients. No statistically significant correlation was observed between pathological response and clinical stage, Gleason score and PSA level (data not shown).

### Outcome

Median follow-up time was 35 months (range, 23–47). In only one patient, the first PSA determination after RP was ⩾0.4 ng ml^−1^. Sixteen (29%) patients received post-operative radiation therapy for positive surgical margins, extraprostatic involvement or PSA relapse. The patient with pathological lymph node involvement started immediate hormonal therapy after surgery.

During the follow-up, 18 (31.6%) patients had a biochemical relapse and 2 had a clinical relapse. None of the 3 patients with pCR relapsed. Among the 18 patients who had a biochemical relapse, 7 had received adjuvant radiation therapy. Conversely, of the total 16 patients who received adjuvant radiation therapy, 7 (43.7%) had a biochemical relapse, a finding that is consistent with the selection of poor prognosis patients for adjuvant radiation therapy. However, the size of the study, the present follow-up and the variations on post-surgical management of the patients limit the possibility of any meaningful results in terms of recurrence rates.

## Discussion

In this study, we report that a combination of weekly D plus a short-term CAB is safe and induces a modest pCR rate in high-risk localised PC. However, the study did not meet the primary end point (pCR rate was 6%) and there was no relationship between response and other risk factors (i.e. Gleason, PSA, T stage), suggesting that the addition of D to hormonal therapy did not have a significant effect on pCR.

Earlier randomised studies of neoadjuvant hormonal therapy showed a lower incidence of positive surgical margins and lymph node metastasis, but without impact on patient outcome. In early reports, the pCR rates ranged from 5 to 15%. However, no relationship between pathological response to neoadjuvant hormonal therapy and patient survival was observed. In studies using short-term neoadjuvant hormonal therapy (3 months, as used in this study), serum PSA did not reach undetectable levels in most patients and pCRs were rarely observed (0–4%), although tumour down-staging and reduction of positive surgical margins were reported (Gleave *et al*, 1996; Prezioso *et al*, 2004; Klotz *et al*, 2005; Pendleton *et al*, 2007; D'Amico *et al* 2008).

Likewise, neoadjuvant D alone has shown activity but not pCRs in high-risk PC patients. In a phase II trial of D administered on a weekly schedule for 6 weeks before RP, pathologic analysis revealed residual carcinoma in all cases. Three patients (11%) had organ-confined disease, and 26 (93%) achieved an undetectable PSA. At a median follow-up of 23 months, 20 patients were disease free with no additional therapy (Dreicer *et al*, 2004). Another group studied 19 patients treated with neoadjuvant weekly D for 6 months and they did not report any pCR. At 26.5 months of follow-up, 81% of patients were free of biochemical relapse (Febbo *et al*, 2005). In another trial, 28 patients were treated with 6 weekly doses of D (40 mg m^−2^) by RP. No pCRs were observed. At a median follow-up of 49.5 months, 12 patients (43%) remained clinically and biochemically free of disease with no additional therapy (Magi-Galluzzi *et al*, 2007). Combination of D with other agents such as mitoxantrone (Garzotto *et al*, 2006) or capecitabine (Friedman *et al*, 2008) has also failed to show pRC.

This trial and a few studies published earlier tested the combination of neoadjuvant D with hormonal therapy in high-risk PC. One group treated 20 patients with a sequential schedule of LHRH analogue until the PSA nadir and then continued hormonal therapy with estramustine and D. They found that one (5%) patient had no residual tumour (pT0) and six (32%) had residual cancer in at least 10% of the surgical specimen. At 53 months of follow-up, 42% of the patients were disease free. In that trial, organ-confined disease (*P*=0.022), residual cancer in ⩽10% of the surgical specimen (*P*=0.007) and no seminal vesicle invasion (*P*=0.001) correlated with DFS (Prayer-Galetti *et al*, 2007). A large phase II multicentre study of 6 months of androgen ablation with weekly D included 72 patients with high-risk PC, with 64 of them completing protocol therapy. Two (3%) pCRs and a 30% biochemical relapse rate at 42.7 months of follow-up were reported (Chi *et al*, 2008).

To the best of our knowledge, this is the first study to explore the feasibility and activity of 3 months of maximum androgen blockade associated with weekly D in high-risk PC before prostatectomy. The 6% of pCR in our study is in the range of the previously reported studies with longer duration therapy with D and hormonal therapy (Prayer-Galetti *et al*, 2007; Chi *et al*, 2008). In the whole series, 47% of patients had pathological organ-confined disease, similar to expected rates with neoadjuvant hormonal therapy alone. We would like to note that 72% of patients with clinically organ-confined disease (T1 or T2) were included. Without any neoadjuvant therapy, 27–35% of patients with these clinical T stages are expected to have pathological organ-confined disease. The rate of positive surgical margins in our series was 35%, similar to the rates expected with neoadjuvant hormonal therapy alone (Gleave *et al*, 1996; Prezioso *et al*, 2004; Klotz *et al*, 2005; Pendleton *et al*, 2007; D'Amico *et al*, 2008). This result further suggests that D added little to hormonal therapy. In our series, only one patient had pathological lymph node involvement. This rate (3.9%) of lymph node disease was considerably lower than what is expected (close to 20%) in extended lymph node dissection in high-risk patients (Heidenreich *et al*, 2007; Burkhard and Studer, 2008). There are several potential explanations for this discrepancy, including lymph node sampling error or inclusion of not truly high-risk patients. The study included patients with either clinical stage T3 disease (28%), or clinical stage T1c or T2 with adverse PSA and/or Gleason score (72%), as detailed in Patients and methods section. We cannot rule out that our population, taken together, had less advanced disease than other reported series because of the selection criteria (other studies included basically cT3/T4 disease) or other variations among different studies. It is also tempting to speculate that the addition of D to hormonal treatment might have had an impact on lymph node status. However, this possibility cannot be addressed in our study and, in fact, other relevant end points (pCRs, surgical margins) do not support this concept. We would like to point out that the fact that the addition of D did not increase the rate of pCR compared with hormonal therapy does not completely rule out a possible benefit in micrometastatic disease and long-term outcome. As mentioned earlier, no relationship between the percentage of pCRs to neoadjuvant hormonal treatment and patient outcome has been reported to date (Gleave *et al*, 1996; Prezioso *et al*, 2004; Klotz *et al*, 2005; Pendleton *et al*, 2007; D'Amico *et al*, 2008). However, in an earlier study of neoadjuvant D and hormone therapy, a correlation between the presence of residual tumour in <10% of the surgical specimen and a better DFS was observed (Prayer-Galetti *et al*, 2007). In our trial, long-term outcome was not objective and the limited sample size as well as the fact that a significant percentage of patients received adjuvant radiation therapy might further limit the correct interpretation of any possible effect on outcome.

Another issue that needs to be considered is the schedule of D used in our and other studies of neoadjuvant therapy. The administration of D every 3 weeks is superior in terms of response and overall survival than weekly D in metastatic hormone-independent PC (Tannock *et al*, 2004). When this trial was designed, these data were not well established and the weekly D schedule was chosen because of its low toxicity profile. However, hormone-naive localised high-risk PC might be a biologically different disease for which the optimal dose and schedule of D, if any, have not been determined yet.

Another unresolved question is the timing of combination therapy. In a pre-clinical PC model, simultaneous androgen deprivation with paclitaxel was more effective than a sequential schedule (Eigl *et al*, 2005). In another preclinical study, immunodeficient mice bearing human LNCaP prostate tumours were treated with D and/or surgical castration applied singly, concurrently or in different sequences. Docetaxel followed by castration improved outcomes in LNCaP PC-bearing mice, compared with simultaneous therapy (Tang *et al*, 2006). The optimal timing and scheduled combination of hormone and chemotherapy have not been clinically tested in PC.

In conclusion, the combination of D and CAB used in our trial failed to meet the primary end point of at least 10% of the pCR rate. Moreover, the percentage of positive margins and the rate of PSA relapse did not differ from the results of the trials with neoadjuvant hormonal therapy alone. Therefore, our data do not support the concept of neoadjuvant D-based chemotherapy in combination with hormonal therapy as a valuable strategy, at least in terms of effects in the primary tumour, in high-risk PC. The results of an ongoing randomised trial (Eastham *et al*, 2003) on neoadjuvant hormonal therapy in combination with D may help to further define the possible role, if any, of this agent in high-risk PC patients.

## Figures and Tables

**Table 1:** Patients characteristics

Total number	57 patients
Median age (years)	68.3 (range, 49–75)
	
*ECOG*	
0	51 (80%)
1	5 (8.4%)
Unknown	1 (0.6%)
	
*Clinical stage*	
T1	11 (19%)
T2	30 (53%)
T3	16 (28%)
	
*Gleason score*	
<6	3 (5%)
7	28 (48%)
8	15 (27%)
9	11 (20%)
	
*PSA (ng ml*^−*1*^)	
>20	15 (26%)
<20	42 (74%)
	
Median PSA (ng ml^−1^)	9.7 (range, 0.6–90.8)
	
*Number of risk factors* a	
1	33 (58%)
2	21 (36%)
3	3 (5%)

Abbreviations: *ECOG*=Eastern Cooperative Oncology Group; PSA=prostate-specific antigen.

a Risk factors: T3, Gleason ⩾7 (4+3), PSA >20 ng ml^−1^.

**Table 2:** Toxicity

**Toxicity**	**Grade 1, *n* (%)**	**Grade 2, *n* (%)**	**Grade 3, *n* (%)**	**Grade 4, *n* (%)**
Anaemia	42 (73)	1 (2)		
Plaquetopenia	4 (7)	1 (2)		
Neutropenia	10 (18)	4 (7)		
Fluid retention	1 (2)			
Alopecia	11 (20)	2 (4)		
Nail toxicity	9 (16)			
Rash	2 (4)	2 (4)		
Mucositis	3 (6)	2 (4)		
Diarrhoea	13 (23)	7 (12)	4 (7)	1(2)
Nausea/vomiting	10 (18)	8 (14)		
Hepatic	10 (18)	12 (21)	7 (12)	
Fatigue	13 (23)	8 (14)		
Neurotoxicity	4 (7)			

**Table 3:** Clinical characteristics of patients with pCR and near pCR (microscopic residual disease) in surgical specimens

**Clinical T stage**	**Preoperative PSA (ng ml^−1^)**	**Gleason score**	**Pathological response**
T1c	9.38	9 (4+5)	pRC
T2a	4.76	8 (4+4)	pRC
T2a	3.18	7 (4+3)	pRC
T3	0.61	7 (4+3)	Near pRC
T3	13	7 (3+4)	Near pRC
T2b	4.3	8 (3+5)	Near pRC
